# Crude protein content in diets associated with intestinal microbiome and metabolome alteration in Huanjiang mini-pigs during different growth stages

**DOI:** 10.3389/fmicb.2024.1398919

**Published:** 2024-04-16

**Authors:** Yating Liu, Md. Abul Kalam Azad, Xichen Zhao, Xiangfeng Kong

**Affiliations:** ^1^Key Laboratory of Agro-Ecological Processes in Subtropical Region, Hunan Provincial Key Laboratory of Animal Nutritional Physiology and Metabolic Process, Institute of Subtropical Agriculture, Chinese Academy of Sciences, Changsha, Hunan, China; ^2^College of Advanced Agricultural Sciences, University of Chinese Academy of Sciences, Beijing, China; ^3^Research Center of Mini-Pig, Huanjiang Observation and Research Station for Karst Ecosystems, Chinese Academy of Sciences, Huanjiang, Guangxi, China

**Keywords:** crude protein, Huanjiang mini-pig, microbiome, metabolome, small intestine

## Abstract

**Introduction:**

Adequate crude protein (CP) content in diets plays a crucial role in the intestinal health of the animal. This study investigated the impacts of CP content in diets on the intestinal microbiome and metabolome profiles in growing Huanjiang mini-pigs.

**Methods:**

A total of 360 pigs with similar body weight (BW) were allocated for three independent feeding trials based on three different BW stages, including (i) 5–10 kg BW, diets consisting of 14, 16, 18, 20, and 22% CP content; (ii) 10–20 kg BW, diets consisting of 12, 14, 16, 18, and 20% CP content; and (iii) 20–30 kg BW, diets consisting of 10, 12, 14, 16, and 18% CP content. These experiments lasted 28, 28, and 26 days, respectively.

**Results:**

The results showed that the Shannon and Simpson indices were decreased (*p* < 0.05) in the ileum of pigs in response to the 14–18% CP compared with the 20% CP content at 5–10 kg BW stage, while diets containing 12 and 14% CP had higher Chao1 (*p* < 0.05) and Shannon (*p* = 0.054) indices compared with 18% CP at 20–30 kg BW stage. Compared with the 20% CP, the diet containing 16% CP displayed an increasing trend (*p* = 0.089) of Firmicutes abundance but had decreased (*p* = 0.056) Actinobacteria abundance in the jejunum at 5–10 kg BW stage. In addition, a diet containing 16% CP had higher *Lactobacillus* abundance in the jejunum and ileum compared with the 18, 20, and 22% CP, while had lower *Sphingomonas* and *Pelomonas* abundances in the jejunum and *Streptococcus* abundance in the ileum compared with the diet containing 22% CP (*p* < 0.05). Diets containing lower CP content altered differential metabolites in the small intestine at the early stage, while higher CP content had less impact.

**Conclusion:**

These findings suggest that a diet containing lower CP content (16% CP) may be an appropriate dietary CP content for 5–10 kg Huanjiang mini-pigs, as 16% CP content in diet has shown beneficial impacts on the intestinal microbiome and metabolome profiles at the early growth stage of pigs.

## Introduction

The development of domestic pig breeding plays a crucial role in the establishment of the agricultural economy of a country. Dietary crude protein (CP) content is one of the most fundamental factors and plays an important role in maintaining the growth and development of pigs. However, high CP contents in diets lead to excessive intake of essential amino acids, and the fermentation of protein in the hindgut impairs intestinal health (Wang et al., 2018). Moreover, inadequate dietary CP content in diets largely affects the intestinal health of pigs. The epithelial morphology of the duodenum and jejunum is impaired with low dietary CP in pigs (Yu et al., 2019). However, reducing CP content in diets could reduce harmful protein fermentation, such as the reduction of ammonia concentration in the intestines of pigs (Pieper et al., 2012). Generally, the National Research Council (NRC) recommends requirements of dietary CP contents in diets for pigs during different growth stages; however, the recommended CP contents could be standardized depending on the geographically challenged conditions and pigs’ genotype (Lee et al., 2022). Therefore, it is necessary to evaluate the optimal dietary CP contents in diets for domestic pig breeds to minimize production costs and establish a better agricultural economy for the pig industry.

The small intestine is mainly associated with the absorption and digestion of proteins and contains undigested or incompletely digested foods, especially proteins and carbohydrates (Portune et al., 2016). The intestinal microbes are associated with food digestion, and various metabolites produced during the digestion processes can be transmitted through intestinal mucosa to affect the host’s body condition (Blachier et al., 2017). Therefore, the proportion of beneficial and pathogenic bacteria and various metabolites produced by intestinal microbes are crucial for maintaining the health of animals, especially during the growing phase. The colonization of host intestinal microbes mostly occurs in piglets at an early age, while the composition of the intestinal microbes of adult pigs is generally stable (Matamoros et al., 2013). Thus, exploring the microbiome and metabolome profiles of pigs during the growing phase is of great significance.

Previous studies have revealed the impacts of high or low CP contents in diets on intestinal microbes at different phases of pigs (Pieper et al., 2012; Yu et al., 2019). Our previous studies systematically evaluated the optimal dietary CP contents in diets for Huanjiang mini-pigs to improve the growth performance at different growth phases (Zhao et al., 2021). The findings indicated that higher CP content in diets could increase the intestinal inflammatory status of growing pigs by activating the TLR-MyD88-NF-κB signaling pathway (Liu et al., 2022). Moreover, lower or higher CP contents in diets had potential native effects on the antioxidant capacity of growing Huanjiang mini-pigs (Liu et al., 2023). However, the impacts of different CP contents in diets on the intestinal microbiome and metabolome profiles of Huanjiang mini-pigs have not been reported yet, and it still remains unknown how dietary CP contents influence the intestinal microbiome and metabolome profiles of pigs during different growth stages. Thus, this study was carried out to the hypothesis that an appropriate CP content in the diet could optimize the microbiome and metabolome composition of Huanjiang mini-pigs based on their growth stages.

## Materials and methods

### Animals, management, and diets

The animal trials were performed at the Huanjiang Observation and Research Station for Karst Ecosystems, Huanjiang, China. Huanjiang mini-pigs (360; half male and half female) with similar body weight (BW) and health status were selected and divided into three corresponding groups for 28-, 28-, and 26-day trials. These three trials were categorized as the 5–10 kg, 10–20 kg, and 20–30 kg BW stages groups after acclimation for 5 days. The 5–10 kg BW stage (Exp. 1) consisted of 220 pigs (28 days of age) with an average BW of 5.32 ± 0.46 kg and were randomly allocated into five CP (14, 16, 18, 20, and 22%) content groups. In the 5–10 kg BW stage, each CP content group consisted of 8–10 pens (2.0 m × 3.0 m) with five pigs in each pen. The 10–20 kg growth stage (Exp. 2) consisted of 84 pigs (60 days of age) with an average BW of 11.27 ± 1.43 kg and were randomly allocated into five CP (12, 14, 16, 18, and 20%) content groups. In the 10–20 kg BW stage, each CP content group consisted of 15–19 pens (1.5 m × 0.6 m), with one pig per pen. The 20–30 kg growth stage (Exp. 3) consisted of 56 pigs (94 days of age) with an average BW of 18.80 ± 2.21 kg and were randomly allocated into five CP (10, 12, 14, 16, and 18%) content groups. In the 20–30 kg BW stage, each CP content group consisted of 11–12 pens (1.5 m × 0.6 m), with one pig per pen.

The experimental pigs received three times (08:00, 14:00, and 20:30) meals per day and had access to feed and water *ad libitum* at all times. All experimental pigs were housed in a well-ventilated piggery with controlled humidity (60 ± 5%) and at 23–25°C temperature. Supplementing CP content in diets met the NRC recommended requirements (National Research Council, 2012) and the Chinese nutrient requirements (Ministry of Agriculture of the People’s Republic of China, 2004) (Supplementary Tables S1–S3). Diet premixes for individual ingredients were formulated using the recommended values by the National Research Council (2012). All pigs were in good health condition, and had no gastrointestinal diseases or any antibiotic exposure prior to the experimental trial.

### Sample collection

Based on the average BW of each experimental pen, one pig from each pen (each CP group had eight pens; total eight pigs) was selected for the 5–10 kg BW stage; and based on the average BW of each CP content group, eight pigs from each group for the 10–20 kg and 20–30 kg BW stages were selected (12 h fasting) for sampling after euthanization by electrical stunning (120 V, 200 Hz; Electric Pig Stunner; Qingdao Jianhua Food Machinery Manufacturing Co. Ltd., Qingdao, China) and exsanguination. Approximately 2 cm of the jejunum (10 cm below the flexure of the duodenum-jejunum) and ileum (10 cm above the ileo-cecal junction) contents were sampled into 1.5 mL sterilized frozen tubes, immediately frozen in liquid nitrogen, and finally preserved at −80°C for bacterial DNA isolation and metabolome analysis.

### Bacterial DNA extraction

The total bacterial genomic DNA from all intestinal samples (jejunum and ileum) was extracted with OMEGA Soil DNA Kit (M5635-02; Omega Bio-Tek, Norcross, GA, United States) following the manufacturer’s instructions and stored at −20°C prior to further analysis. The quantity and quality of the extracted DNA were confirmed using a NanoDrop NC2000 spectrophotometer (Thermo Fisher Scientific, Waltham, MA, United States) and agarose gel electrophoresis, respectively.

### 16S rRNA gene amplicon sequencing

The polymerase chain reaction (PCR) amplification of the bacterial 16S rRNA genes with V3–V4 region was performed using the universal forward primer 338F (5′-ACTCCTACGGGAGG CAGCA-3′) and the reverse primer 806R (5′-GGACTACHV GGGTWTCTAAT-3′). Sample-specific 7-bp barcodes were incorporated into the primers for multiplex sequencing. The PCR reaction components contained 5.00 μL of buffer (5×), 0.25 μL of Fast pfu DNA Polymerase (5.00 U/μL), 2.00 μL (2.50 mM) of dNTPs, 1.00 μL (10 μM) of each forward and reverse primer, 1.00 μL of DNA template, and 14.75 μL of ddH_2_O. Thermal cycling conditions for PCR consisted of initial denaturation at 98°C for 5 min, followed by 25 cycles consisting of denaturation at 98°C for 30 s, annealing at 53°C for 30 s, and extension at 72°C for 45 s, with a final extension of 5 min at 72°C. The amplicons of PCR were purified with the Vazyme VAHTSTM DNA Clean Beads (Vazyme, Nanjing, China) and quantified using the Quant-iT PicoGreen dsDNA Assay Kit (Invitrogen, Carlsbad, CA, United States), following the manufacture’s protocols. After the individual quantification step, purified amplicons were pooled in an equal amount, and the pair-end (2 × 250 bp) sequencing was performed on an Illumina NovaSeq platform with NovaSeq 6,000 SP Reagent Kit (500 cycles) by the Shanghai Personal Biotechnology Co. Ltd. (Shanghai, China).

### Sequence analysis

Microbiome bioinformatics analyses were performed using the QIIME2 v.4 with slight modifications according to the official tutorials.^1^ Briefly, raw sequence data were demultiplexed using the demux plugin, followed by primers cutting with the cutadapt plugin. Sequences were then quality filtered, denoised, merged, and chimera removed with DADA2 plugin. The non-singleton amplicon sequence variants (ASVs) were combined with mafft and performed to construct a phylogeny with fasttree2. The alpha-diversity metrics (including Chao1, Observed_species, Shannon, Simpson, Faith’s PD, Pielou’s evenness, and Good’s coverage) and beta-diversity metrics (including weighted UniFrac, unweighted UniFrac, Jaccard distance, and Bray–Curtis dissimilarity) were determined with the diversity plugin. Taxonomy was assigned to ASVs using the classify-sklearn naïve Bayes taxonomy classifier in a feature-classifier plugin against the Green genes Databases.

### Bioinformatics analysis

Sequence data analyses were performed using the QIIME2 and R packages (v3.2.0). The ASV-level alpha-diversity metrics, including Chao1 richness estimator, Observed_species, Shannon index, and Simpson index, were calculated using the ASV table in QIIME2. Beta-diversity analysis was used to identify the microbial community structure variations among samples using the unweighted UniFrac distance metrics and visualized through principal coordinate analysis (PCoA). Principal component analysis (PCA) was conducted based on the compositional profiles at the genus level. The linear discriminant analysis effect size (LEfSe) was performed to detect differentially abundant taxa among different groups using the default parameters. Phylogenetic investigation of communities by reconstruction of unobserved state (PICRUSt) was used to characterize the functional capacity of the small intestinal microbiota of pigs.

### UPLC–MS analysis for intestinal metabolites

The metabolite contents in the jejunum and ileum of Huanjiang mini-pigs were determined using a non-targeted metabolomics approach with the UPLC-HDMS. The metabolomics procedures included sample preparation, metabolite separation and detection, data preprocessing, and statistical analysis.

For metabolite identification, approximately 25 mg of each sample was weighed into a 2-mL EP tube and then added 500 μL extract solution [acetonitrile: methanol: water = 2:2:1 (v/v), with the isotopically-labeled internal standard mixture] to the EP tube. After 30 s of vortexing, the mixed samples were homogenized at 35 Hz for 4 min and sonicated in an ice-water bath for 5 min. The homogenization and sonication cycles were repeated three times. Then the samples were incubated for 1 h at −40°C and centrifuged at 12,000 × *g* for 15 min at 4°C. The resulting supernatants were filtered through a 0.22-μm membrane and transferred to fresh glass vials for further analysis. The quality control (QC) sample was obtained by mixing an equal aliquot of the supernatants from all samples.

An ultra-performance liquid chromatography (UPLC) system (Vanquish, Thermo Fisher Scientific, Waltham, MA, United States) with a UPLC BEH Amide column (2.10 × 100 mm, 1.70 μm) coupled with Q Exactive HFX mass spectrometer (Orbitrap MS, Thermo Fisher Scientific, Waltham, MA, United States) was used to perform LC–MS/MS analyses. The mobile phase A contained 25 mmol/L ammonium acetate and 25 mmol/L ammonia hydroxide in water, and the mobile phase B contained acetonitrile. The injection volume was 3 μL, and the temperature of the auto-sampler was set at 4°C. To acquire MS/MS spectra on an information-dependent acquisition (IDA) mode, the QE HFX mass spectrometer was used for its ability in the control of the acquisition software (Xcalibur, Thermo Fisher Scientific, Waltham, MA, United States). In this mode, the acquisition software continuously evaluated the full scan of the MS spectrum. The conditions for ESI source were set as follows: sheath gas flow rate 30 Arb, Aux gas flow rate 25 Arb, capillary temperature 350°C, full MS resolution 60,000, MS/MS resolution 7500, collision energy 10/30/60 in NCE mode, and spray voltage 3.60 kV (positive ion mode) or −3.20 kV (negative ion mode), respectively.

For peak detection, extraction, alignment, and integration, obtained raw data were converted into mzXML format by ProteoWizard and then processed with an in-house program, which was developed using R and based on XCMS. The metabolites were annotated using an in-house MS2 (secondary mass spectrometry) database (BiotreeDB v2.1). The value of the cutoff was 0.3. The PCA and orthogonal partial least squares discriminant analysis (OPLS-DA) were established by the SIMCA software v.16.0.2 (Sartorius Stedim Data Analytics AB, Umea, Sweden) to visualize the distinction and detect differential metabolites among different CP content groups. Moreover, the Kyoto Encyclopedia of Genes and Genomes (KEGG) and MetaboAnalyst 5.0 were used for pathway analysis.

### Statistical analysis

All experimental data were statistically analyzed by a one-way analysis of variance using SPSS v.26.0 software (SPSS Inc., Chicago, IL, United States) package. All data were checked for normal distribution, and then Tukey *post-hoc* test was subjected to comparative analyses among different groups. The individual pigs were considered the experimental unit. Data are presented as means ± standard error of the mean (SEM). The significance and a trend toward differences were considered as *p* < 0.05 and 0.05 ≤ *p* < 0.10, respectively. The GraphPad Prism 8.0 (San Diego, CA, United States) was used for processing images.

## Results

### Effects of different CP content in diets on the small intestinal microbiota community

**Figure 1:**
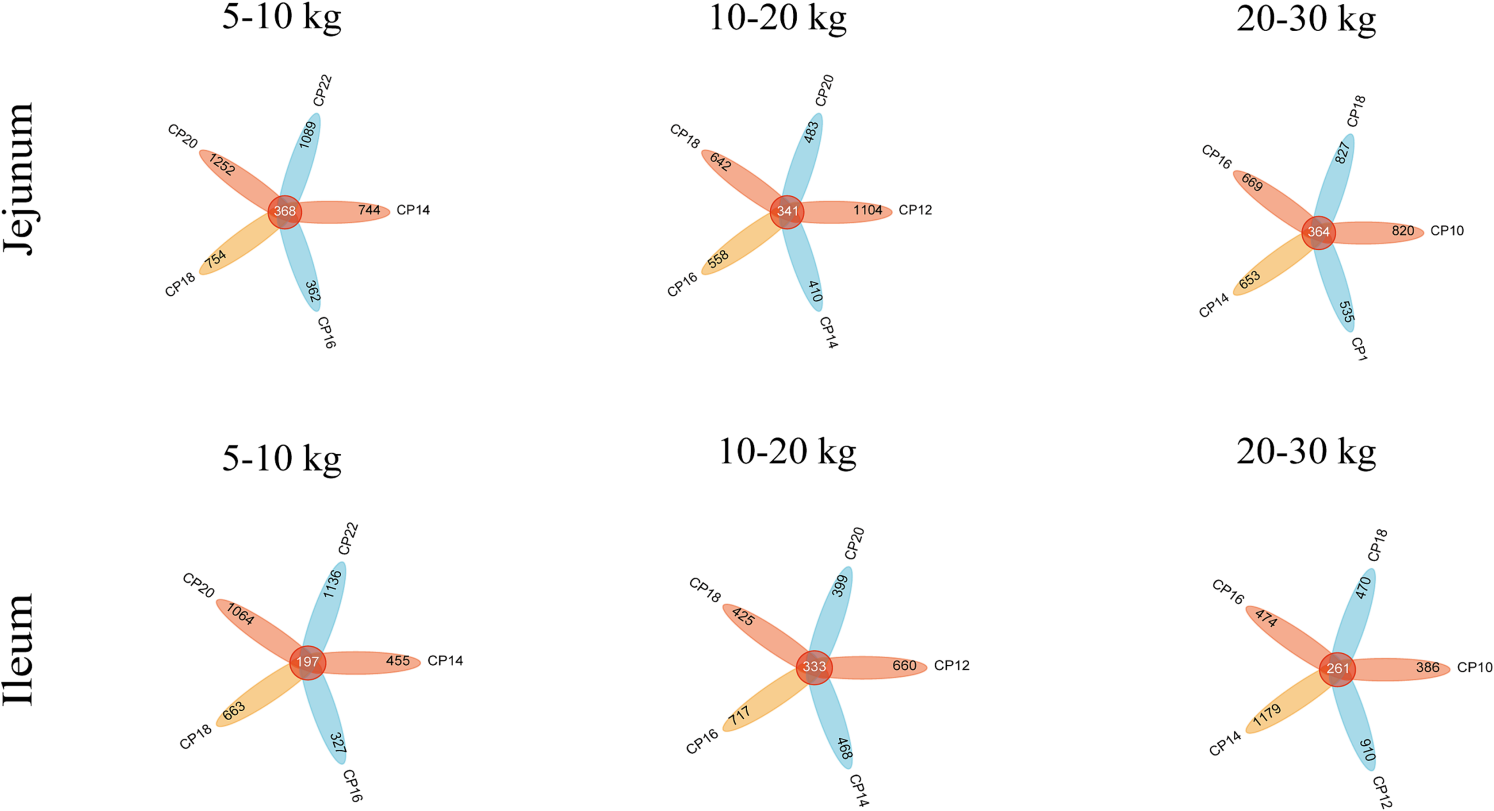
Effects of different crude protein (CP) content in diets on the microbiota community in the small intestine of Huanjiang mini-pigs during different body weight (BW) stages.

Effects of different CP content in diets on the small intestinal microbiota community of Huanjiang mini-pigs are presented in Figure 1. Based on the high-throughput sequencing, a total of 5,541,169 raw sequences were generated from 30 jejunal and 29 ileal samples at 5–10 kg BW stage. After a series of processing, including filtering, denoising, and removing chimeras, a total of 3,923,641 high-quality sequences were obtained and aggregated into ASVs. Based on 97% sequencing similarity, a total of 4,569 bacterial ASVs were annotated in the jejunum, and 368 bacterial ASVs were common among different CP content groups, whereas 3,842 bacterial ASVs were annotated in the ileum, and 197 bacterial ASVs were common among different CP content groups.

At 10–20 kg BW stage, a total of 7,428,973 raw sequences were generated from 39 jejunal and 39 ileal samples of Huanjiang mini-pigs. After filtering, denoising, and removing chimeras, a total of 5,281,664 high-quality sequences were obtained and aggregated into ASVs (Figure 1). A total of 3,538 bacterial ASVs were annotated in the jejunum of which 341 were common bacterial ASVs, whereas 3,002 bacterial ASVs were annotated in the ileum of which 333 were common bacterial ASVs based on 97% sequencing similarity.

At 20–30 kg BW stage, a total of 7,234,867 raw sequences were generated from 35 jejunal and 35 ileal samples of Huanjiang mini-pigs, of which 5,044,147 high-quality sequences were obtained and aggregated into ASVs. Based on 97% similarity, 3,868 bacterial ASVs and 364 common ASVs were annotated in the jejunum, and 3,680 bacterial ASVs and 261 common ASVs were annotated in the ileum.

### Effects of different CP content in diets on the small intestinal microbiota diversity

The small intestinal microbiota alpha-diversity, including Chao 1, Shannon, Simpson, and Observed_species indices of Huanjiang mini-pigs during different growth stages, are presented in Table 1. At 5–10 kg BW stage, there were no significant differences (*p* > 0.05) in the jejunum alpha-diversity indices among the five CP content groups. The 14, 16, and 18% CP contents in diets decreased (*p* < 0.05) the Shannon and Simpson indices in the ileum of pigs compared with the 20% CP content in the diet. At 10–20 kg BW stage, different CP content in diets had no impact (*p* > 0.05) in the jejunum and ileum alpha-diversity indices. At 20–30 kg BW stage, 12 and 14% CP contents in diets increased (*p* < 0.05) the Chao 1 index in the ileum compared with the 10, 16, and 18% CP contents. The 12 and 14% CP contents in diets displayed an increasing trend (*p* = 0.054) in the Shannon index in the ileum of pigs compared with the 18% CP content. Moreover, 14% CP content in the diet increased (*p* < 0.05) the Observed_species index in the ileum of pigs compared with the other CP contents in diets. However, there were no significant differences (*p* > 0.05) in the jejunum alpha-diversity indices among different CP content at 20–30 kg BW stage.

**Table 1:** Effects of different crude protein (CP) content in diets on the small intestinal microbiota alpha-diversity of Huanjiang mini-pigs during different body weight (BW) stages.

Items	Dietary CP contents (%)					*p*-values
Jejunum						
5–10 kg BW	14	16	18	20	22	
Chao1	603.53 ± 132.35	503.12 ± 63.89	710.23 ± 77.25	776.74 ± 158.53	747.44 ± 154.38	0.514
Shannon	4.53 ± 0.75	3.71 ± 0.47	4.93 ± 0.46	5.53 ± 0.60	4.52 ± 0.79	0.370
Simpson	0.79 ± 0.10	0.71 ± 0.06	0.83 ± 0.07	0.89 ± 0.06	0.77 ± 0.11	0.633
Observed_species	489.28 ± 99.01	418 ± 48.33	578.53 ± 58.95	633.73 ± 120.34	597.78 ± 123.27	0.540
10–20 kg BW	12	14	16	18	20	
Chao1	511.79 ± 92.62	344.67 ± 35.18	448.79 ± 64.54	473.62 ± 41.31	444.20 ± 56.01	0.404
Shannon	4.27 ± 0.44	3.23 ± 0.41	3.35 ± 0.44	4.53 ± 0.32	4.00 ± 0.39	0.108
Simpson	0.77 ± 0.06	0.64 ± 0.07	0.67 ± 0.07	0.82 ± 0.05	0.75 ± 0.05	0.210
Observed_species	432.04 ± 72.43	296.43 ± 29.00	369.88 ± 49.41	397.53 ± 30.41	375.41 ± 46.86	0.377
20–30 kg BW	10	12	14	16	18	
Chao1	603.01 ± 46.88	424.54 ± 64.11	451.75 ± 33.75	524.83 ± 65.39	539.89 ± 61.08	0.187
Shannon	4.92 ± 0.25	3.89 ± 0.44	4.10 ± 0.32	4.55 ± 0.33	4.17 ± 0.24	0.201
Simpson	0.89 ± 0.02	0.76 ± 0.07	0.81 ± 0.04	0.84 ± 0.04	0.82 ± 0.02	0.293
Observed_species	492.03 ± 37.53	350.54 ± 47.48	376.29 ± 25.47	437.81 ± 53.63	438.53 ± 43.69	0.170
Ileum						
5–10 kg BW	14	16	18	20	22	
Chao1	406.05 ± 30.95	391.87 ± 46.44	541.74 ± 105.12	689.76 ± 115.98	675.89 ± 204.76	0.188
Shannon	3.08 ± 0.38^b^	3.25 ± 0.44^b^	3.45 ± 0.49^b^	5.07 ± 0.38^a^	4.50 ± 0.76^ab^	0.030
Simpson	0.59 ± 0.08^b^	0.68 ± 0.07^b^	0.66 ± 0.08^b^	0.88 ± 0.03^a^	0.79 ± 0.07^ab^	0.036
Observed_species	340.47 ± 24.63	336.95 ± 37.28	435.08 ± 81.96	552.98 ± 82.71	555.04 ± 169.63	0.225
10–20 kg BW	12	14	16	18	20	
Chao1	362.50 ± 46.12	387.84 ± 51.15	528.35 ± 65.76	408.55 ± 48.43	438.4 ± 49.13	0.222
Shannon	3.14 ± 0.46	3.21 ± 0.53	3.78 ± 0.41	3.32 ± 0.36	3.54 ± 0.48	0.854
Simpson	0.60 ± 0.08	0.61 ± 0.08	0.69 ± 0.07	0.64 ± 0.07	0.65 ± 0.08	0.908
Observed_species	313.33 ± 40.52	332.58 ± 44.16	442.15 ± 52.04	348.90 ± 39.97	372.26 ± 42.12	0.288
20–30 kg BW	10	12	14	16	18	
Chao1	451.20 ± 38.12^b^	611.14 ± 84.24^a^	617.68 ± 54.98^a^	410.32 ± 37.36^b^	314.47 ± 42.92^b^	0.001
Shannon	3.93 ± 0.31	4.29 ± 0.65	4.50 ± 0.30	3.33 ± 0.37	2.85 ± 0.44	0.054
Simpson	0.76 ± 0.05	0.74 ± 0.09	0.82 ± 0.04	0.66 ± 0.06	0.60 ± 0.08	0.199
Observed_species	297.71 ± 43.18^b^	320.99 ± 40.06^b^	482.96 ± 52.10^a^	328.04 ± 43.55^b^	339.83 ± 32.25^b^	0.035

Data are presented as means ± SEM (*n* = 6–8 per group). ^a,b^Different superscript letters within the same row indicate significant differences among the groups (*p* < 0.05). BW, body weight; CP, crude protein.

**Figure 2:**
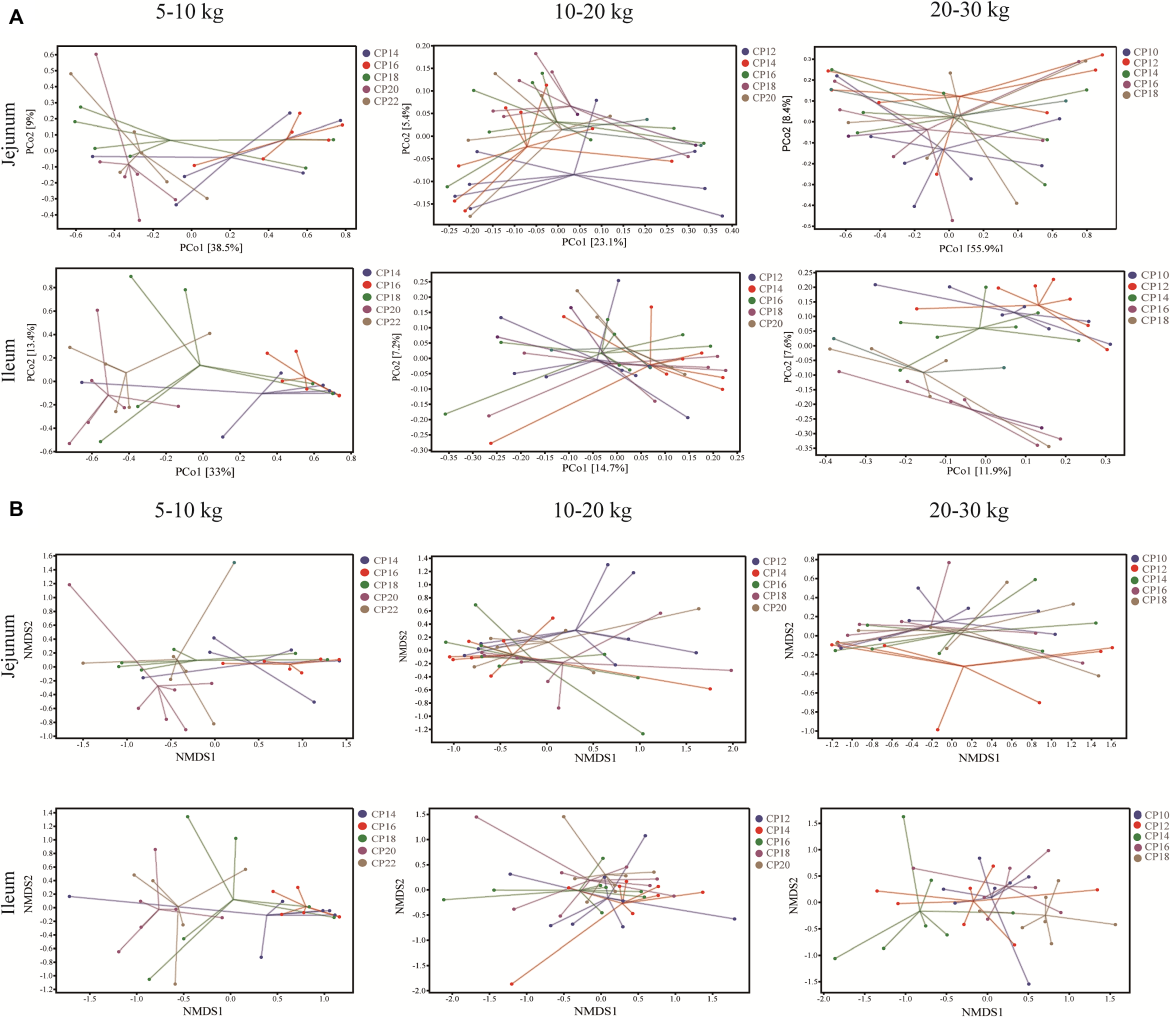
Effects of different crude protein (CP) content in diets on the small intestinal microbiota beta-diversity of Huanjiang mini-pigs during different growth stages. Principal coordinate analysis (PCoA) score plots of the microbiota in the jejunum and ileum of pigs during different body weight (BW) stages **(A)** and non-metric multidimensional scaling (NMDS) ordinations score plots based on Bray-Curtis distance metric of microbiota in the jejunum and ileum of pigs during different BW stages **(B)**.

Effects of different CP content in diets on the small intestinal microbiota beta-diversity of Huanjiang mini-pigs are presented in Figure 2. The PCoA analysis showed that there were significant separations of the microbial community structure in the jejunum and ileum of pigs between 14 and 16% CP contents and 20 and 22% CP contents at 5–10 kg BW stage, as well as the microbial community structure in the ileum of pigs at 20–30 kg BW stage (Figure 2A). Further non-metric multidimensional scaling (NMDS) analysis was used to assess intergroup microbial community structure distance among different CP content in diets of the different BW groups. The microbial community structure had significant separations in the jejunum and ileum at 5–10 kg BW stage, in the jejunum at 10–20 kg BW stage, as well as in the jejunum and ileum at 20–30 kg BW stage (Figure 2B).

### Effects of different CP content in diets on the small intestinal microbial community composition

**Figure 3:**
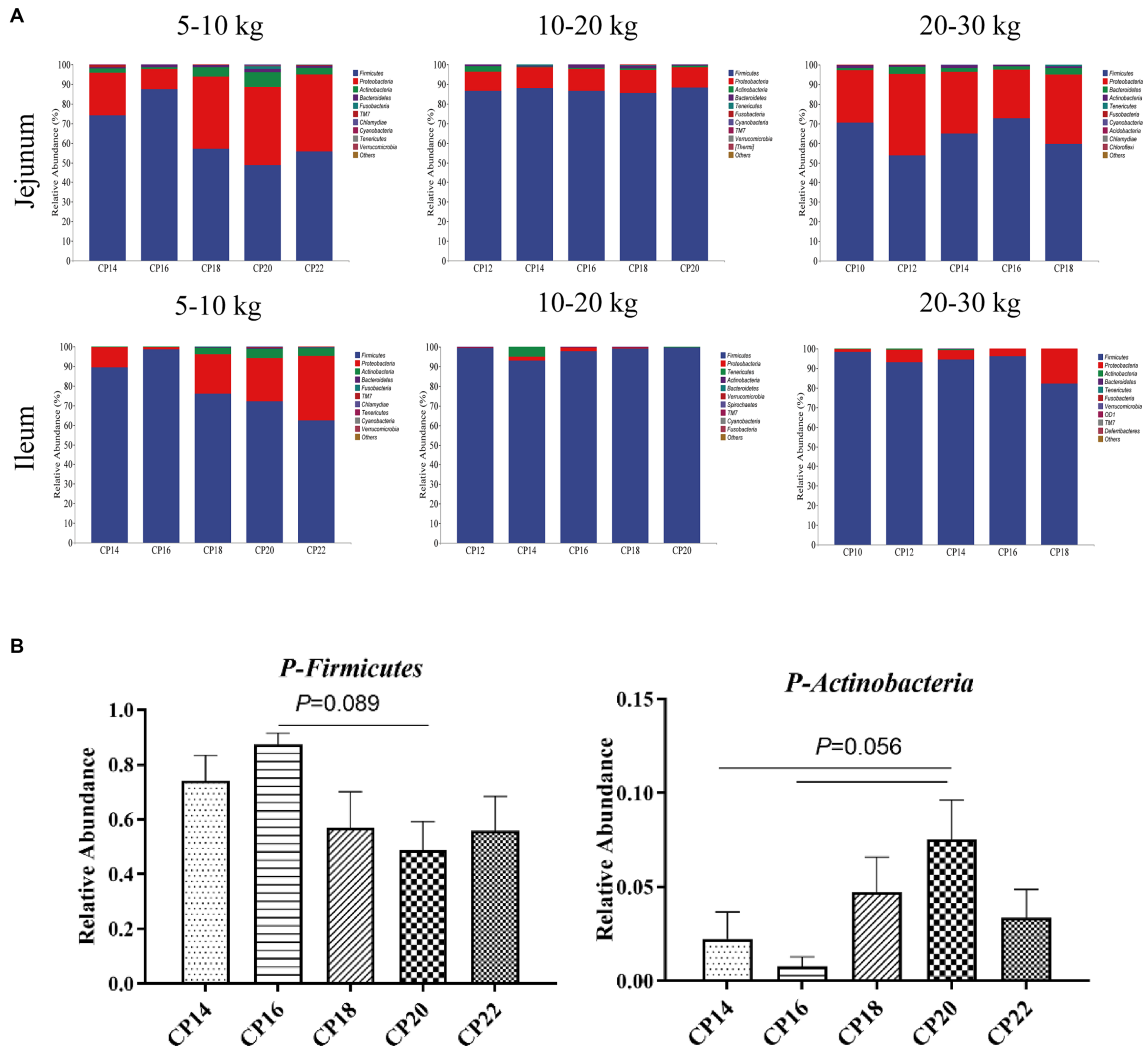
Effects of different crude protein (CP) content in diets on the small intestinal microbiota community composition **(A)** at the phylum level during different body weight (BW) stages and taxonomic differences in the jejunum **(B)** at 5–10 kg BW of Huanjiang mini-pigs.

**Figure 4:**
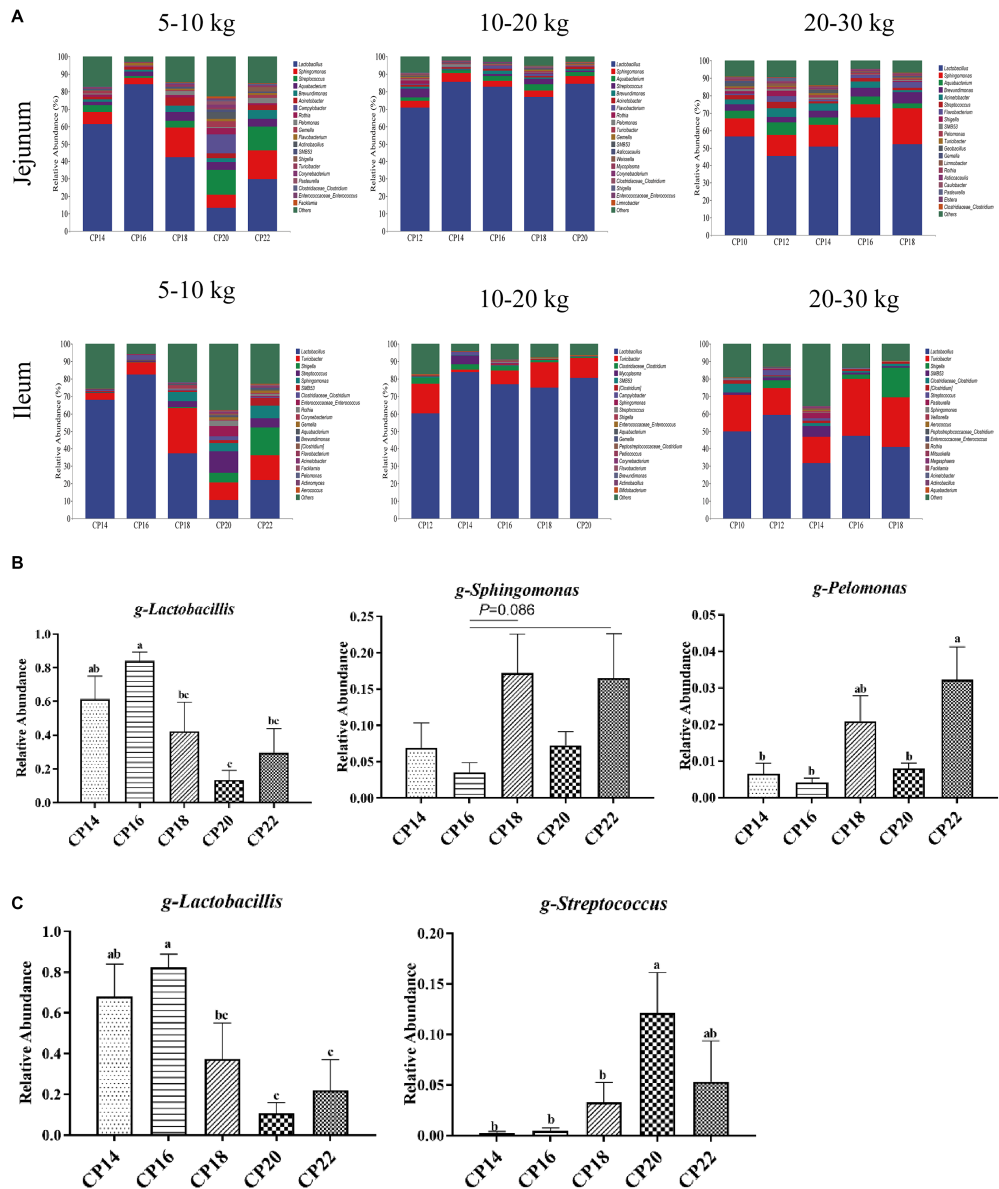
Effects of different crude protein (CP) content in diets on the small intestinal microbiota community composition **(A)** of Huanjiang mini-pigs at the genus level during different body weight (BW) stages. Taxonomic differences in the jejunum **(B)** and ileum **(C)** at 5–10 kg BW of Huanjiang mini-pigs. a–c Values with different lowercase letters means significant difference (*p* < 0.05).

Based on 97% sequence similarity, the top 10 bacterial phyla and top 20 bacterial genera in the jejunum and ileum of Huanjiang mini-pigs during different BW stages were identified (Figures 3, 4). At 5–10 kg BW stage, Firmicutes (74.09, 87.41, 56.99, 48.72, and 55.81%), Proteobacteria (21.70, 10.43, 37.21, 39.82, and 39.04%), Actinobacteria (2.24, 0.78, 4.73, 7.53, and 3.37%), and Bacteroidetes (0.44, 1.30, 0.96, 1.83, and 1.23%) were the top dominant phyla in the jejunum of the 14, 16, 18, 20 and 22% CP content groups, respectively, accounting more than 90% of the total bacterial phyla (Figure 3A). Additionally, 16% CP content in the diet displayed an increasing trend (*p* = 0.089) of Firmicutes abundance, while 14 and 16% CP contents in diets showed a decreasing trend (*p* = 0.056) of Actinobacteria abundance in the jejunum compared with the 20% CP (Figure 3B). In the ileum, Firmicutes (89.33, 98.69, 76.00, 72.18, and 62.47%), Proteobacteria (10.40, 1.07, 19.98, 21.90, and 32.91%), and Actinobacteria (0.15, 0.12, 3.34, 4.70, and 3.73%) were the top three dominant phyla of the 14, 16, 18, 20, and 22% CP content groups, accounting more than 90% of the total bacterial phyla (Figure 3A). However, there were no significant differences (*p* > 0.05) in bacterial phyla abundances in the ileum among different CP content groups at 5–10 kg BW stage.

At 10–20 kg BW stage, the dominant phyla in the jejunum of the 12, 14, 16, 18, and 20% CP content groups were Firmicutes (86.57, 87.96, 86.76, 85.64, and 88.23%) and Proteobacteria (9.88, 10.78, 10.91, 11.57, and 10.48%), whereas in the ileum were Firmicutes (99.36, 92.93, 97.86, 99.27, and 99.58%) and Proteobacteria (0.43, 2.18, 1.85, 0.51, and 0.25%) (Figure 3A). There were no significant differences (*p* > 0.05) in the abundances of bacterial phyla in the jejunum and ileum at 10–20 kg BW stage.

At 20–30 kg BW stage, Firmicutes (70.38, 53.87, 65.03, 72.82, and 59.72%), Proteobacteria (26.94, 41.51, 31.34, 24.69, and 35.27%), and Bacteroidetes (1.06, 3.49, 1.88, 1.77, and 3.36%) in the jejunum and Firmicutes (98.32, 93.17, 94.44, 96.18, and 82.31%) and Proteobacteria (1.51, 6.40, 4.78, 3.69, and 17.62%) in the ileum were the top abundant bacterial phyla of the 10, 12, 14, 16, and 18% CP content groups (Figure 3A). However, there were no significant differences (*p* > 0.05) in the abundances of bacterial phyla in the jejunum and ileum at 20–30 kg BW stage.

Effects of different CP content in diets on the small intestinal microbial community composition of Huanjiang mini-pigs during different BW stages at the genus level are presented in Figure 4. At 5–10 kg BW stage, *Lactobacillus* (61.38, 84.12, 42.24, 13.50, and 29.75%), *Sphingomonas* (6.92, 3.58, 17.22, 7.29, and 16.54%), and *Streptococcus* (3.72, 1.26, 3.68, 14.25, and 13.61%) were the most dominant genera in the jejunum of the 14, 16, 18, 20, and 22% CP content groups. In addition, bacterial genera in the jejunum with >1% relative abundances were *Aquabacterium*, *Brevundimonas*, and *Acinetobacter* (Figure 4A). The diet consisting of 16% CP increased (*p* < 0.05) the relative abundance of *Lactobacillus* in the jejunum of pigs compared with the 18–22% CP contents, while 16% CP content in the diet displayed a decreasing trend (*p* = 0.086) of *Sphingomonas* abundance in the jejunum of pigs compared with the 18 and 22% CP contents. The 14, 16, and 20% CP contents in diets decreased (*p* < 0.05) the *Pelomonas* abundance in the jejunum of pigs compared with the 22% CP (Figure 4B). The dominant bacterial genera in the ileum of the 14, 16, 18, 20, and 22% CP content groups were *Lactobacillus* (68.03, 82.49, 37.33, 10.65, and 22.04%), *Turicibacter* (3.85, 7.05, 26.04, 9.91, and 14.07%), and *Shigella* (0.19, 0.15, 0.36, 5.64, and 15.93%) at 5–10 kg BW stage (Figure 4A). The 14–16% CP contents in diets increased (*p* < 0.05) the *Lactobacillus* abundance in the ileum of pigs compared with the 20–22% CP contents, while 14–18% CP contents in diets decreased (*p* < 0.05) the *Streptococcus* abundance compared with the 20% CP content (Figure 4C). At 10–20 kg BW stage, the most dominant genera in the jejunum of the 12, 14, 16, 18, and 20% CP content groups were *Lactobacillus* (70.85, 85.51, 82.72, 76.92, and 84.51%), *Sphingomonas* (3.80, 5.05, 3.29, 3.52, and 4.39%), and *Aquabacterium* (2.03, 1.60, 2.75, 3.66, and 2.13%), whereas in the ileum were *Lactobacillus* (60.28, 83.73, 76.83, 74.92, and 80.60%), *Turicibacter* (16.88, 1.42, 7.81, 14.42, and 11.29%), and *Clostridiaceae*-*Clostridium* (3.26, 3.23, 2.43, 1.19, and 0.47%) (Figure 4A). At 20–30 kg BW stage, *Lactobacillus* (56.62, 45.48, 50.57, 67.52, and 52.07%), *Streptococcus* (10.27, 11.77, 12.55, 7.35, and 20.54%), *Aquabacterium* (4.34, 7.35, 4.35, 4.53, and 2.93%), and *Brevundimonas* (3.67, 3.07, 3.94, 5.07, and 6.29%) were the most abundant genera in the jejunum, and *Lactobacillus* (49.91, 59.44, 31.72, 47.27, and 41.05%), *Turicibacter* (20.90, 15.21, 15.03, 32.62, and 28.37%), and *Shigella* (0, 4.46, 0, 2.69, and 16.80%) were the most abundant genera in the ileum of the 12, 14, 16, 18, and 20% CP content groups (Figure 4A). There were no significant differences (*p* > 0.05) in the abundances of the small intestinal genera at 10–20 kg and 20–30 kg BW stages.

### Effects of different CP content in diets on metabolic capability profiles of the small intestinal microbiota

**Figure 5:**
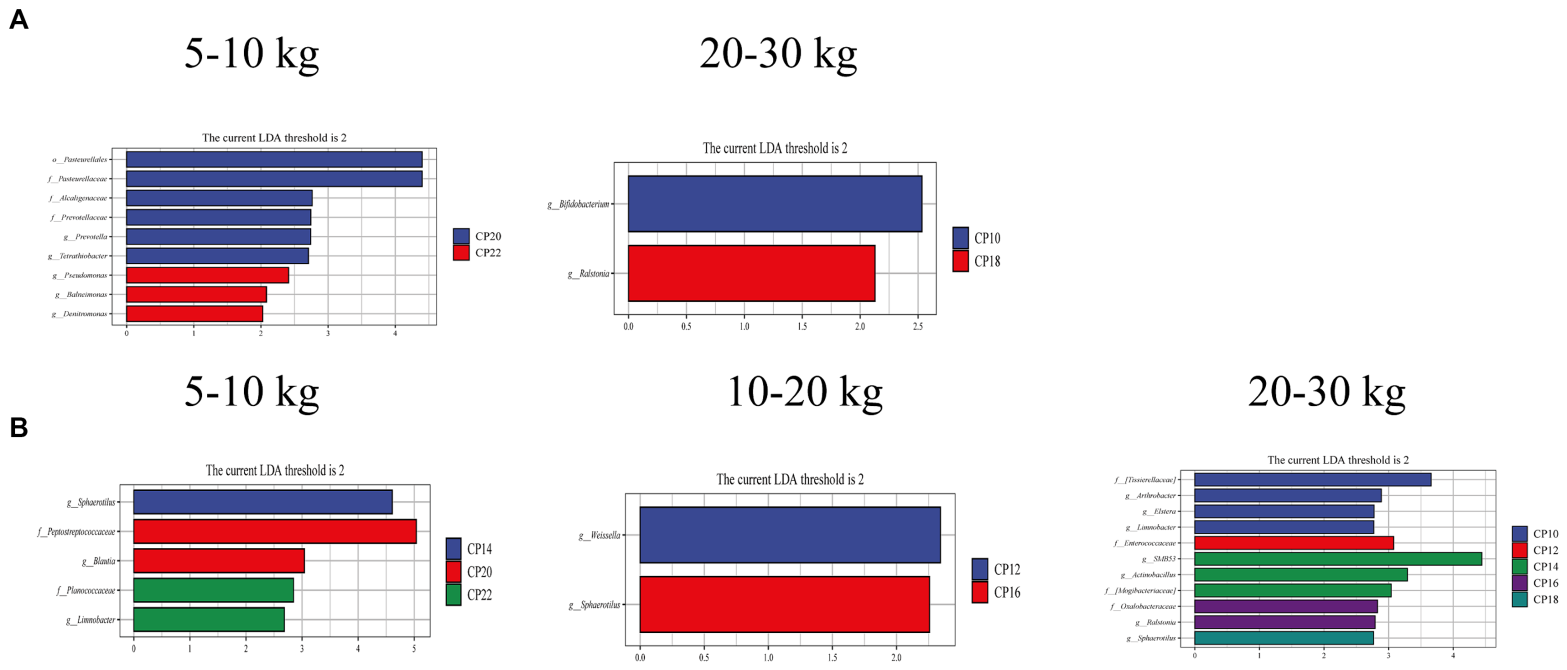
Analysis of taxonomic abundance of microbiota using linear discriminant analysis effect size (LEfSe) analysis (LDA score ≥ 2, *p* < 0.05) in the jejunum **(A)** and ileum **(B)** of Huanjiang mini-pigs during different body weight (BW) stages.

Different CP content in diets on the small intestinal metabolic capacity of Huanjiang mini-pigs during different BW stages are presented in Figure 5. The LEfSe analysis revealed that 20% CP content in the diet enriched the *Prevotella* and *Tetrathiobacter*, while 22% CP content in the diet enriched the *Pseudomonas*, *Balneimonas*, and *Denitromonas* differential marker genera in the jejunum of pigs at 5–10 kg BW stage (Figure 5A). In the ileum, the differential marker genera *Sphaerotilus* (14% CP), *Blautia* (20% CP), and *Limnobacter* (22% CP) were enriched at 5–10 kg BW stage (Figure 5B). At 10–20 kg BW stage, the differential marker genera *Weissella* (12% CP) and *Sphaerotilus* (16% CP) were enriched in the ileum (Figures 5A,B). At 20–30 kg BW stage, the differential marker genera *Bifidobacterium* (10% CP) and *Ralstonia* (18% CP) in the jejunum and *Elstera* (10% CP), *Limnobacter* (10% CP), *Enterococcaceae* (12% CP), *SMB53*, *Actinobacillus* (14% CP), *Ralstonia* (16% CP), and *Sphaerotilus* (18% CP) in the ileum were enriched in the different CP content groups (Figures 5A,B).

**Figure 6:**
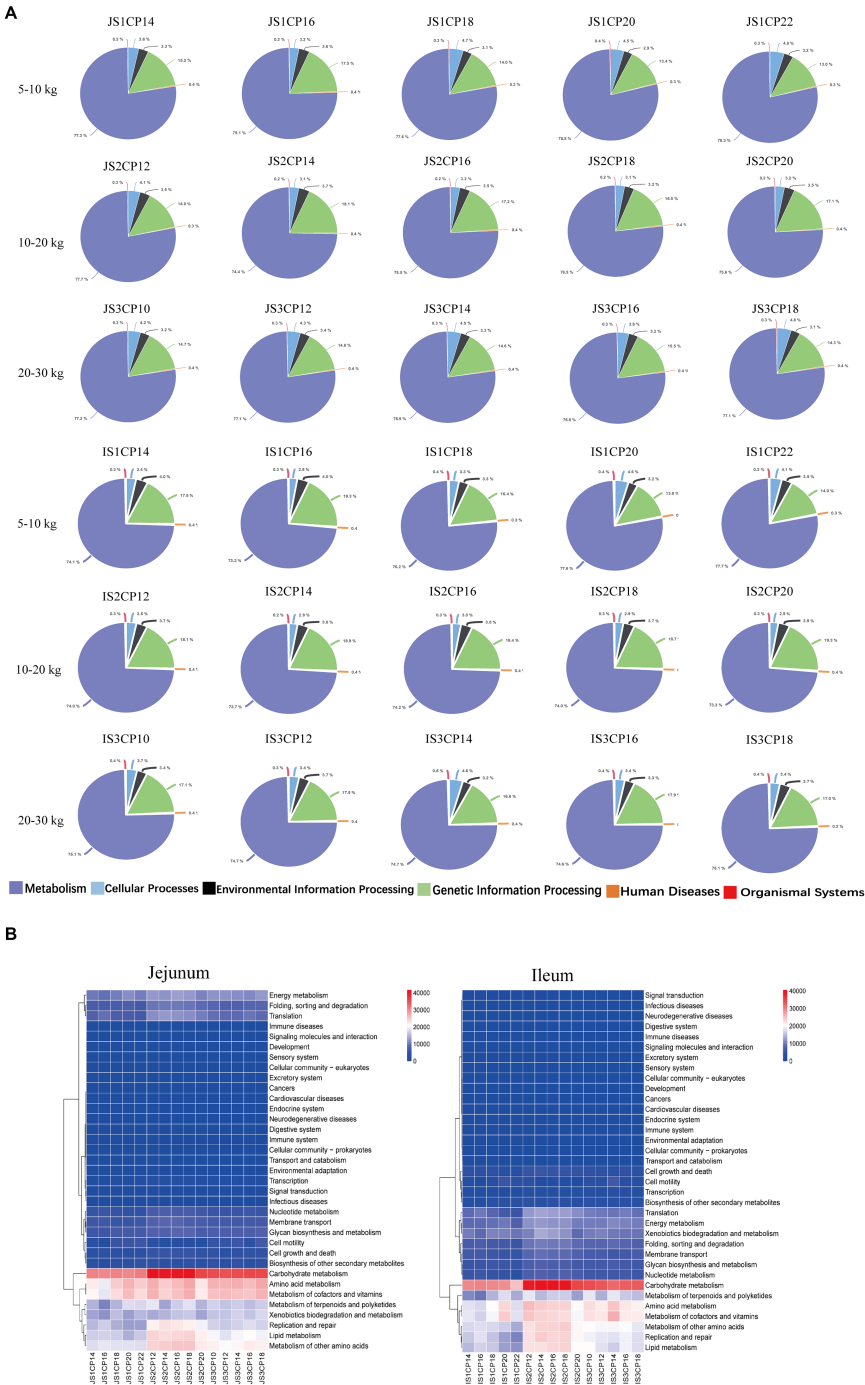
Predictive metagenomics showing differences in the function among different CP content in diets of Huanjiang mini-pigs during different body weight (BW) stages. Metabolic pathways with different functional categories using the level 1 **(A)** analysis and level 2 **(B)** PICRUST analysis.

The impacts of different CP content in diets of Huanjiang mini-pigs during different BW stages on the small intestinal microbiota predictive function are shown in Figure 6. The level 1 PICRUST functional prediction analysis was used to compare functional enrichment of microbiota among the five CP content groups, and metabolic pathways were divided into six functional categories (including cellular processes, environmental information processing, genetic information processing, human diseases, metabolism, and organismal systems) (Figure 6A). In addition, 45 differential gene functions were observed among different CP content groups by level 2 PICRUST function prediction (Figure 6B). At 5–10 kg BW stage, there were significant changes in the majority pathways related to cell growth and death, replication and repair, infectious diseases, energy metabolism, terpenoid and polyketide metabolism, and endocrine system and immune system-related functions or pathways in the jejunum. In the ileum, the pathways belonged to cell motility, replication and repair, translation, infectious diseases, amino acid metabolism, biosynthesis of other secondary metabolites, and lipid metabolism and digestive system related functions or pathways. At 10–20 kg BW stage, there were no significant differences in the microbial functional pathways in the jejunum and ileum of the different CP content groups. At 20–30 kg BW stage, the pathways related to cell community-prokaryote and carbohydrate metabolism were enriched in the jejunum, whereas glycan biosynthesis and metabolism and cofactors and vitamins metabolism were enriched in the ileum of pigs.

### Effects of different CP content in diets on the small intestinal metabolome profile

In order to reveal the effects of different CP content in diets on the small intestinal metabolome profile of Huanjiang mini-pigs during different BW stages, metabolites extracted from jejunal and ileal samples were detected by UPLC-QTOF/MS. A total of 12,494 peaks were obtained from the positive ion mode (ESI^+^) and 9,940 peaks from the negative ion mode (ESI^−^) of LC–MS. After denoising, filtering, and data standardization, 8,586 (ESI^+^) and 6,979 (ESI^−^) valid peaks were obtained from the positive and negative ion modes, respectively. Finally, 845 (ESI^+^) and 334 (ESI^−^) differential metabolite signals were detected after matching with the local database (Shanghai Biotree Biomedical Technology Co. Ltd., Shanghai, China) (Supplementary Tables S4–S7).

Combined with our previous findings on immune- and antioxidant-related data (Liu et al., 2022, 2023), metabolomics analysis was considered by comparing the highest, lowest, and best dietary CP content in diets at each BW stage. The comparison groups included 14% vs. 20% and 20% vs. 22% CP contents for the 5–10 kg BW stage, 12% vs. 16% and 16% vs. 20% CP contents for the 10–20 kg BW stage, and 10% vs. 12% and 12% vs. 18% CP contents for the 20–30 kg BW stage. Considering the higher differential metabolite signals (845 vs. 334; ESI^+^ vs. ESI^−^), this study mainly analyzed the data under the ESI ^+^ mode.

**Figure 7:**
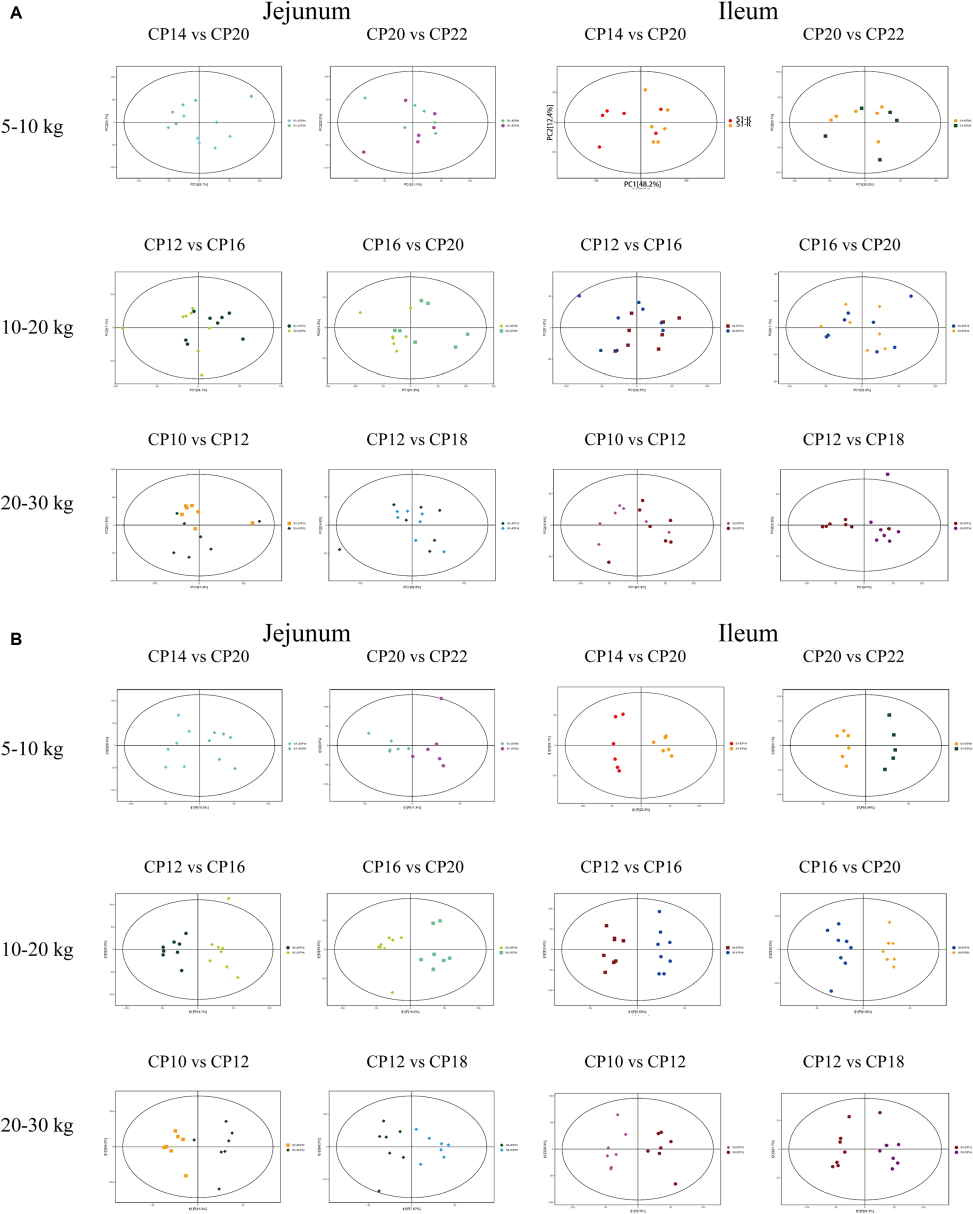
The PCA analysis **(A)** and score plots of OPLS-DA **(B)** analysis of the small intestinal metabolites of different dietary crude protein (CP) content in diets of Huanjiang mini-pigs during different body weight (BW) stages.

Firstly, metabolite data in the ESI^+^ mode was visualized using an unsupervised multivariate data analysis method (PCA). As shown in Figure 7A, there were no visible differences between the groups, indicating that there were no significant differences in metabolome profiles between different CP content groups. To maximize the visualization of the changes in metabolome profiles between the different CP content groups, the data collected from the ESI^+^ mode were processed using the supervised multivariate data analysis method (OPLS-DA). As shown in Figure 7B and Supplementary Figure S1, there were significant visible cluster separation effects between groups with different dietary CP content groups, indicating that different CP content in diets significantly changed the small intestinal metabolome pattern of Huanjiang mini-pigs during different BW stages.

### Effects of different CP content in diets on differential metabolites in the small intestinal contents

**Figure 8:**
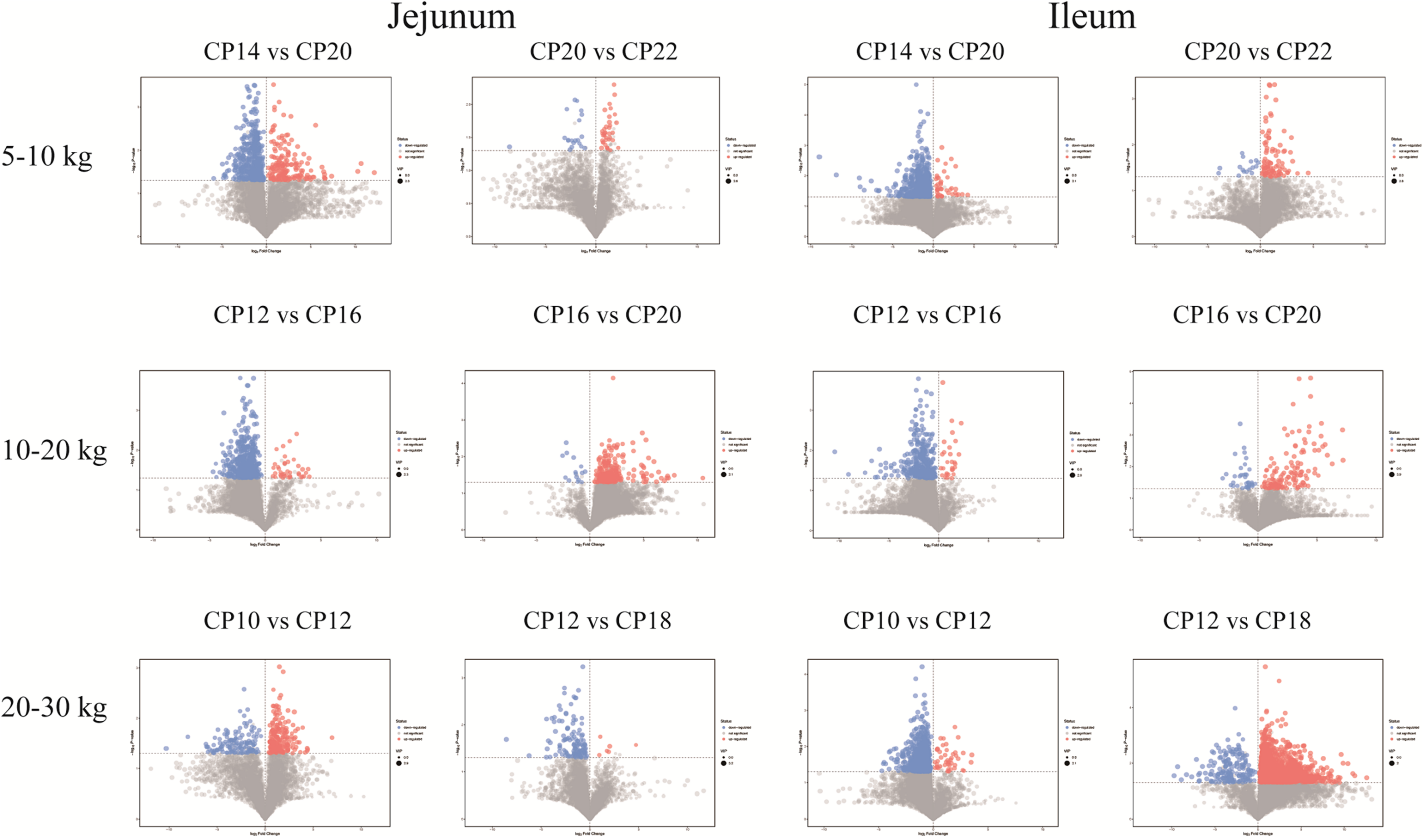
Volcano plots of the small intestinal metabolites based on the non-target metabolomics. Each symbol represents the identified metabolite, orange color represents significantly up-regulated differential metabolites, blue color represents significantly down-regulated differential metabolites, and gray color represents the metabolites those were not differed.

Metabolites from different CP content groups were screened and identified based on parameter criteria of VIP > 1.0 and *p* < 0.05 to screen differential metabolites. Fifty-seven differential metabolites (increased 16 and decreased 41) were identified in the jejunum of the 14% CP content group compared to the 20% CP content group at 5–10 kg BW stage (Figure 8). The differential metabolites mainly included lipids and lipid-like molecules, organic acids and derivatives, and organic heterocyclic compounds. Six differential metabolites (increased three and decreased three) were identified in the jejunum of the 22% CP content group compared to the 20% CP group. These metabolites mainly included amines, amino acids, and organic oxygen compounds. In addition, 111 differential metabolites (increased three and decreased 108) were identified in the ileum of the 14% CP content group compared to the 20% CP group at 5–10 kg BW stage. The differential metabolites mainly included organic acids and derivatives, organic heterocyclic compounds, and lipids and lipid-like molecules. Furthermore, 11 differential metabolites (increased two and decreased nine) were identified in the ileum of the 22% CP content group compared to the 20% CP group at 5–10 kg BW stage. The differential metabolites mainly included organic acids and derivatives, as well as lipids and lipid-like molecules.

At 10–20 kg BW stage, 42 differential metabolites (increased four and decreased 38) were identified in the jejunum of the 12% CP content group compared to the 16% CP content group. These differential metabolites mainly included lipids and lipid-like molecules, organic acids and derivatives, organic heterocyclic compounds, and benzenes. In addition, 34 differential metabolites (all metabolites were decreased) were identified in the jejunum of the 20% CP content group compared to the 16% CP content group. These differential metabolites mainly included lipid and lipid-like molecules, phenylpropane and polyketide, and benzene. A total of 40 differential metabolites (increased two and decreased 38) were identified in the ileum of the 12% CP content group compared to the 16% CP content group. These metabolites mainly included lipid and lipid-like molecules, organic acids and derivatives, organic heterocyclic compounds, and benzene. Moreover, 14 differential metabolites (increased three and decreased 11) were identified in the ileum of the 20% CP content group compared to the 16% CP content group, and the differential metabolites mainly included lipids and lipid-like molecules (Figure 8).

At 20–30 kg BW stage, 23 differential metabolites (increased six and decreased 17) were identified in the jejunum of the 10% CP content group compared to the 12% CP content group. The differential metabolites mainly included lipids and lipid-like molecules, organic acids, and derivatives. In addition, 15 significantly increased differential metabolites were identified in the jejunum of the 18% CP content group compared to the 12% CP content group. The differential metabolites mainly included organic acids and derivatives and organic heterocyclic compounds. A total of 84 differential metabolites (increased six and decreased 78) were identified in the ileum of the 10% CP content group compared to the 12% CP content group. The differential metabolites mainly included organic heterocyclic compounds, organic acids and derivatives, lipids and lipid-like molecules, and benzenes. Furthermore, 227 differential metabolites (increased 20 and decreased 207) were identified in the ileum of the 18% CP content group compared to the 12% CP content group. The differential metabolites mainly included organic heterocyclic compounds, organic acids and derivatives, and lipids and lipid-like molecules (Figure 8).

### Effects of different CP content in diets on the intestinal metabolome metabolism pathways

**Figure 9:**
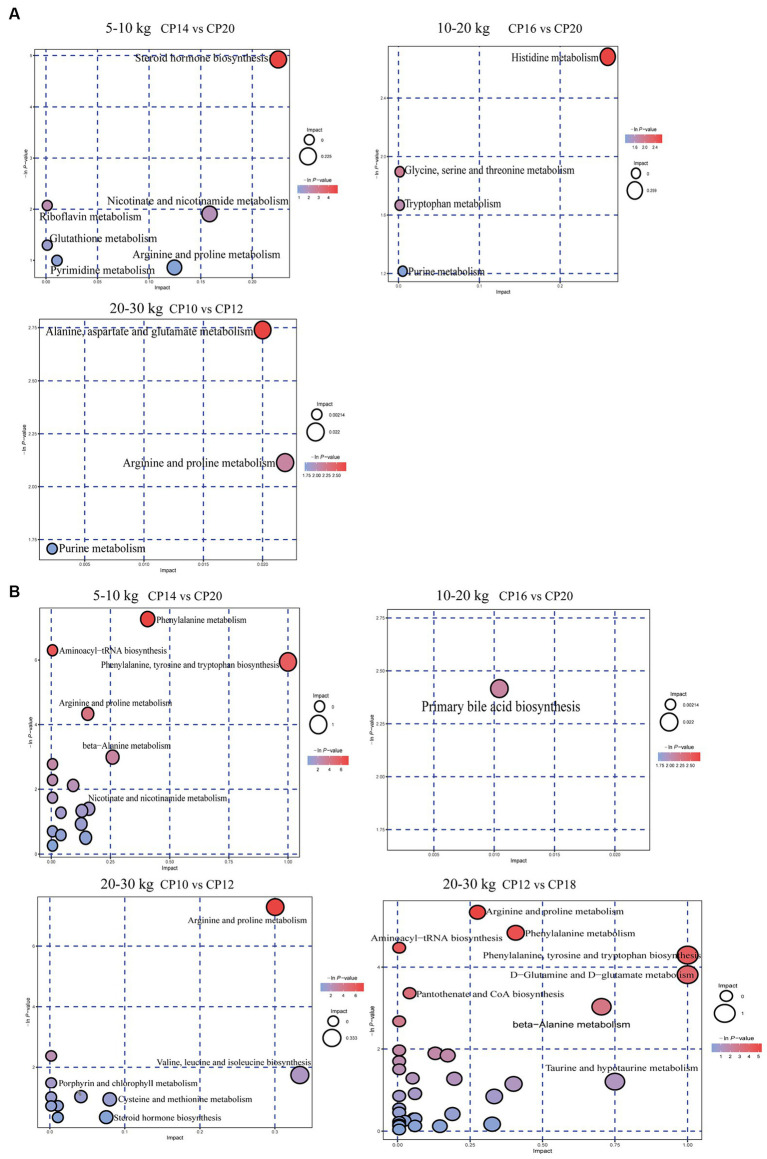
Effects of different crude protein (CP) content in diets on metabolic pathways in the jejunum **(A)** and ileum **(B)** of Huanjiang mini-pigs during different body weight (BW) stages.

The KEGG analysis was used to further analyze the metabolism pathways and metabolite marker of differential metabolites between the high and low CP content in diets and the optimal CP content in the diet (Figure 9). At 5–10 kg BW stage, the steroid hormone biosynthesis pathway was enriched in the jejunum of the 14% CP content group compared to the 20% CP content group, which was related to the four detected steroid differential metabolites. However, no differential metabolic pathway was detected in the jejunum of the 22% CP content group compared to the 20% CP content group (Figure 9A). Moreover, phenylalanine metabolism, aminoacyl-tRNA biosynthesis, phenylalanine/tyrosine/tryptophan biosynthesis, arginine/proline metabolism, and β-alanine metabolism pathways were enriched in the ileum of the 14% CP content group compared to the 20% CP content group, which were associated with 10 amino acids and phytohormone differential metabolites detected. However, no differential metabolite pathways were detected in the ileum of the 20% vs. 22% CP content groups (Figure 9B).

At 10–20 kg BW stage, the histidine metabolism pathway was enriched in the jejunum of the 16% CP content group compared to the 20% CP content group (Figure 9A). In addition, the primary bile acid biosynthesis pathway was enriched in the ileum of the 16% CP content group compared with the 20% CP content group, and it was associated with the detected 25-hydroxycholesterol differential metabolite (Figure 9B). However, there was no pathway enrichment in the jejunum and ileum between the 12 and 16% CP content groups.

At 20–30 kg BW stage, alanine, aspartate, and glutamate metabolic pathways were enriched in the jejunum of the 10% CP content group compared to the 12% CP content group, which were associated with differential metabolites of argininosuccinic acid (Figure 9A). Arginine and proline metabolism, and aminoacyl-tRNA biosynthesis pathways were enriched in the ileum of the 10% CP content group compared to the 12% CP content group, which were associated with six amino acids differential metabolites detected. Moreover, arginine and proline metabolism, phenylalanine metabolism, aminoacyl-tRNA biosynthesis, phenylalanine, tyrosine and tryptophan biosynthesis, d-glutamine and d-glutamate metabolism, pantothenic acid and CoA biosynthesis, *β*-alanine metabolism, and nitrogen metabolism pathways were enriched in the ileum of the 12% CP content group compared to the 18% CP content group. These pathways were associated with 17 amino acids, phytohormones, and carbonyl compounds (Figure 9B). However, there was no metabolic pathway enrichment in the jejunum between the 12 and 18% CP content groups.

## Discussion

The intestinal microbiome and metabolome play an important role in human and animal health. Diet is one of the main factors affecting the intestinal microbiome composition (Ijaz et al., 2018). Dietary CP is a major macronutrient in diets and contributes to maintain the health of animals by regulating the micro-ecological balance and metabolite production in the small intestine. Thus, this study investigated the effects of different CP content in diets on the small intestinal microbiome and metabolome profiles of Huanjiang mini-pigs during different growth stages. The results showed that the optimal CP content in the diet affected the immunity and antioxidant capacity of Huanjiang mini-pigs by regulating the abundant microbiota composition of the small intestine and their amino acid-related metabolic activities.

The intestinal microbiota regulates the gastrointestinal health and function of the host. The higher microbial diversity is beneficial for intestinal homeostasis and the overall health of the host. The Chao1 and Observed_species indices indicate microbial richness, while the Shannon and Simpson indices indicate microbial diversity. In the present study, 20% CP content in the diet increased the Shannon and Simpson indices in the ileum at 5–10 kg BW stage, while 12 and 14% CP contents in diets had higher microbial richness and diversity in the ileum at 20–30 kg BW stage. These findings indicate that 20% CP content in the diet at 5–10 kg BW stage and 12–14% CP contents in diets at 20–30 kg BW stage were more effective in contributing to intestinal homeostasis of piglets. However, different CP content in diets at 10–20 kg BW stage had no impact on the microbial diversity of piglets. The findings were consistent with a previous study, which reported that a reduction of 3% of CP content in diets does not affect microbial richness and diversity (Zhou et al., 2016). Further beta-diversity analysis showed that different dietary CP content influenced the overall microbiota structure in the small intestine of Huanjing mini-pigs during 5–10 and 20–30 kg BW stages.

Firmicutes and Proteobacteria were the most dominant phyla in the jejunum and ileum of Huanjiang mini-pigs during different BW stages, which was different from previous studies (Wang X. et al., 2019; Hou et al., 2021). It has been reported that an increase in the Firmicutes to Bacteroidetes ratio promotes lipid deposition (Wang X.X. et al., 2019) and may be associated with metabolic diseases, such as obesity. We found that 16% CP content in the diet displayed an increasing trend of the Firmicutes abundance in the jejunum compared to the 20% CP content; however, there was no significant difference in the relative abundance of Bacteroidetes in the jejunum among different CP content in diets. Moreover, 14 and 16% CP contents in diets displayed a decreasing trend of Actinobacteria abundance in the jejunum compared to the 20% CP content. *Bifidobacterium* belongs to the Actinobacteria phylum, a potential probiotic that promotes body health and can inhibit colonization by intestinal pathogens (Barba-Vidal et al., 2017) and modulate intestinal immune responses (Kandasamy et al., 2014). However, it has been found that feeding the pigs with a lower CP content in the diet for 120 days increased the *Bifidobacterium* abundance in the jejunum and ileum (Zhang et al., 2020). The inconsistency of our results might be related to the test pigs’ breed and the age in the present study, which was different, and the small intestinal microbiota composition was affected differently. A higher CP content in the diet (CP > 20%) affects the intestinal structure and barrier function, thereby affecting intestinal health, which ultimately leads to an increased rate of diarrhea in piglets (Xia et al., 2022). Previously, we found the diarrhea index was increased in the Huanjiang mini-pigs at 5–10 kg BW stage with a higher CP content in the diet (20% CP) compared to the lower CP content (14–16% CP) (Liu et al., 2022), which may be related to the lower Firmicutes abundance and the higher Proteobacteria abundance in the small intestine of pigs. However, no significant differences were observed in the small intestinal microbiota among different CP content groups during 10–20 kg and 20–30 kg BW stages, which may be because of the small intestinal maturation of Huanjiang mini-pigs during the latter two BW stages.

At the genus level, *Lactobacillus* is one of the major genera in the small intestine of growing pigs. *Lactobacillus* and *Bifidobacteria* ferment carbohydrates into lactic acid and improve intestinal health (Kleerebezem et al., 2003; Fukuda et al., 2011). In the present study, 16% CP content in the diet increased the *Lactobacillus* abundance in the jejunum and ileum of pigs compared to the 20 and 22% CP contents, indicating that moderate protein restriction might increase beneficial bacteria in the small intestine and optimize the structure of the small intestinal microbiota. *Streptococcus* is also a member of lactic acid bacteria and participates in amino acid utilization (Neis et al., 2015), but some of these bacterial species are potential pathogens that adversely affect body health (Fortomaris et al., 2006; Chen et al., 2018). We found that the 14, 16, and 18% CP contents in diets lowered the relative abundance of *Streptococcus* in the ileum compared to the 20% CP content at 5–10 BW stage, which may be related to their insufficient protein substrates required for fermentation. In addition, 16% CP content in the diet also lowered the relative abundance of *Sphingomonas* than the 18 and 22% CP contents, whereas *Sphingomonas* was also lower in the 14, 16, and 20% CP content groups than in the 22% CP content group. *Sphingomonas* and *Pelomonas* are both harmful bacteria, indicating that excessive dietary CP content in diets could increase the relative abundance of harmful bacteria in the small intestine of pigs.

Intestinal microbiota can produce abundant metabolites, which participate in the intestinal physiological function and modulate the metabolic activities of the host (Schroeder and Bäckhed, 2016). In addition, intestinal microbiota-derived metabolites are the key host-microbiota interaction intermediates products and have various physiological functions to maintain the host’s health (Koh and Bäckhed, 2020). It has been demonstrated that dietary intervention significantly affects the composition and abundance of the intestinal microbiota (Cameron-Smith et al., 2020). Through functional prediction of the gut microbiota, it was found that a low-protein content in diets increased the gut microbial metabolic activity in pigs because the gut microbiota genes with different CP content in diets were mainly enriched in “metabolic pathways” (Wang et al., 2020). In the present study, different CP content in diets revealed several differential metabolite differences in the small intestinal sites. For instance, compared with the 14% CP content in the diet at 5–10 kg BW stage, differential metabolites related to lipids and lipid-like molecules, of which four steroidal metabolites were changed, eventually affecting the steroid hormone biosynthesis pathway in the jejunum when diets consisted of 20% CP. Compared to the 14% CP content in the diet, 20% CP content in the diet enhanced 111 differential metabolites in the ileum, most of which were amino acids. These dozens of differential metabolites were involved in phenylalanine metabolism, aminoacyl-tRNA biosynthesis, phenylalanine/tyrosine/tryptophan biosynthesis, arginine and proline metabolism, and β-alanine metabolism pathways, and there were differences in metabolites produced by different intestinal segments under the same comparative method.

In addition to the intestinal tract, metabolites can also be affected by sex, and a lower CP content in the diet has a greater impact on sow metabolism (Tao et al., 2021). However, no differential metabolic pathways were detected in the jejunum and ileum of pigs when the pigs were feeding a diet containing 20% CP compared to the pigs fed a diet containing 22% CP in the present study, possibly because of their small differences in protein content. Moreover, at 10–20 kg BW stage, the histidine metabolism in the jejunum and primary bile acid biosynthesis pathway in the ileum were enriched when the pigs were fed a diet containing 16% CP compared to the 20% CP. Moreover, several pathways were detected related to amino acid metabolism at 20–30 kg BW stage, especially when the 12% CP content was compared with the 18% CP content; multiple amino acid metabolism pathways, such as arginine and proline metabolism, phenylalanine metabolism, aminoacyl-tRNA biosynthesis, and phenylalanine/tyrosine/tryptophan biosynthesis were affected in the ileum. These findings suggest that lower CP content in diets had better effects on beneficial intestinal metabolite enrichment at the early growth stage of Huanjiang mini-pigs, which also supports the intestinal microbiota modulation of young pigs in the present study.

## Conclusion

Based on the findings obtained in the present study, lower CP content in diets (especially 16% CP content in the diet) showed beneficial effects on the intestinal health of piglets by increasing the relative abundances of beneficial bacteria (i.e., Firmicutes and *Lactobacillus*) and decreasing the pathogenic bacteria (i.e., *Sphingomonas* and *Pelomonas*) in the small intestine of Huanjiang mini-pigs at the early growth stage (5–10 kg BW). Moreover, lower CP content in diets was also beneficial for intestinal metabolome profiles, particularly amino acid metabolism in the small intestine of pigs. Although higher CP content in diets showed the potential risk for piglets at the early growth stage; however, the potential risk was diminished after maturing the gut of piglets. These findings suggest the optimal CP content of 16% in the diet for Huanjiang mini-pigs at the early growth stage (5–10 kg BW).

## Data availability statement

The datasets presented in this study can be found in online repositories. The names of the repository/repositories and accession number(s) can be found at the Science Data Bank under accession number https://doi.org/10.57760/sciencedb.16749 and Supplementary Tables S4–S7 under accession number https://figshare.com/articles/dataset/Supplementary_Tables_S4-7_zip/25587057.

## Ethics statement

The study was conducted according to the Chinese guidelines for animal welfare and experimental protocols and was approved by the Animal Care and Use Committee of the Institute of Subtropical Agriculture, Chinese Academy of Sciences (No. ISA-2019-4-29). The study was conducted in accordance with the local legislation and institutional requirements.

## Author contributions

YL: Formal analysis, Investigation, Visualization, Writing – original draft, Writing – review & editing. MA: Formal analysis, Investigation, Methodology, Writing – original draft, Writing – review & editing. XZ: Formal analysis, Investigation, Methodology, Writing – review & editing. XK: Conceptualization, Funding acquisition, Project administration, Supervision, Validation, Writing – review & editing.

## Footnotes

^1^https://docs.qiime2.org/2019.4/tutorials/
