# Supplementary material for: Crude protein content in diets associated with intestinal microbiome and metabolome alteration in Huanjiang mini-pigs during different growth stages
Source: Front Microbiol. 2024 Apr 16;15:1398919. doi: 10.3389/fmicb.2024.1398919 (PMC11058986; doi:10.3389/fmicb.2024.1398919)
Supplement: Supplementary file 1 [file Data_Sheet_1.DOCX]

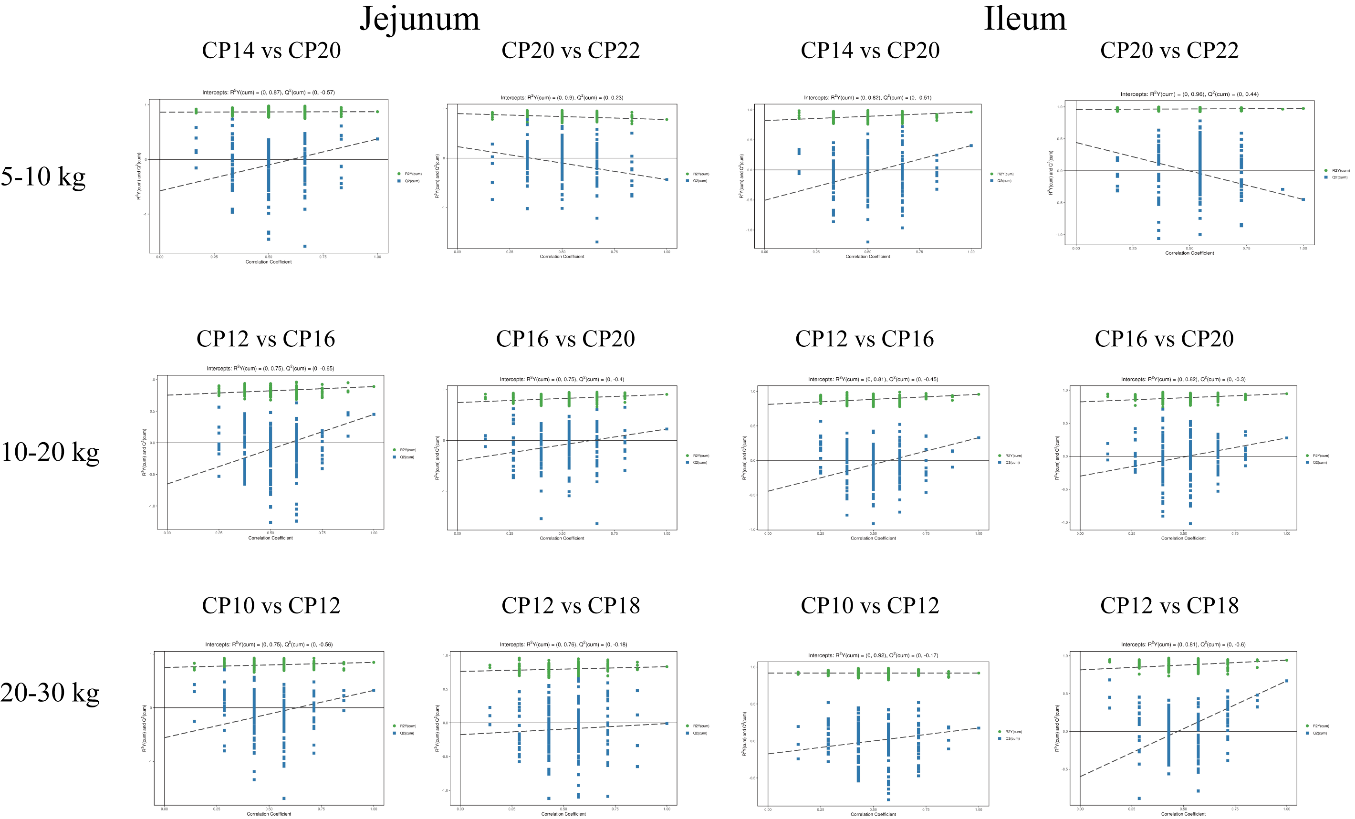


**Figure S1** The OPLS-DA permutation plot of the small intestinal metabolites of different dietary crude protein (CP) levels of Huanjiang mini-pigs.
