# Supplementary material for: Crude protein content in diets associated with intestinal microbiome and metabolome alteration in Huanjiang mini-pigs during different growth stages
Source: Front Microbiol. 2024 Apr 16;15:1398919. doi: 10.3389/fmicb.2024.1398919 (PMC11058986; doi:10.3389/fmicb.2024.1398919)
Supplement: Supplementary file 2 [file Table_1.DOCX]

**Table S1** Ingredients and composition of the basal diet (air-dried, 5−10 kg growth stage)

| **Items** | **Dietary CP levels (%)** | | | | |
| --- | --- | --- | --- | --- | --- |
|  | **14** | **16** | **18** | **20** | **22** |
| **Ingredients, %** |  |  |  |  |  |
| Corn | 61.20 | 56.20 | 51.80 | 47.50 | 42.70 |
| Soybean meal (43.9%) | 5.70 | 10.10 | 13.20 | 15.60 | 18.80 |
| Fish meal | 2.00 | 2.00 | 2.00 | 2.00 | 2.00 |
| Wheat bran | 9.00 | 9.00 | 9.00 | 9.00 | 9.00 |
| Whey powder (85% lactose) | 10.00 | 10.00 | 10.00 | 10.00 | 10.00 |
| Extruded full-fat soybean | 5.00 | 5.00 | 5.00 | 5.00 | 5.00 |
| Soy protein concentrate | 1.00 | 2.10 | 4.00 | 6.30 | 8.20 |
| Corn oil | 0.40 | 0.20 | - | - | - |
| L-Lys (98.5%) | 1.10 | 0.90 | 0.75 | 0.55 | 0.40 |
| DL-Met (99%) | 0.20 | 0.15 | 0.15 | 0.10 | 0.10 |
| L-Thr (99%) | 0.40 | 0.35 | 0.25 | 0.20 | 0.10 |
| L-Trp (99%) | 0.10 | 0.10 | 0.05 | 0.05 | - |
| Dicalcium phosphate | 1.40 | 1.40 | 1.30 | 1.20 | 1.20 |
| Premix^1^ | 2.50 | 2.50 | 2.50 | 2.50 | 2.50 |
| Total | 100.00 | 100.00 | 100.00 | 100.00 | 100.00 |
| **Nutrient levels^2^** |  |  |  |  |  |
| ME, MJ/kg | 13.73 | 13.69 | 13.68 | 13.72 | 13.74 |
| CP | 14.09 | 16.10 | 18.10 | 20.07 | 22.08 |
| SID Lys | 1.36 | 1.34 | 1.36 | 1.34 | 1.36 |
| SID Met | 0.39 | 0.37 | 0.39 | 0.37 | 0.40 |
| SID Thr | 0.76 | 0.79 | 0.77 | 0.80 | 0.77 |
| SID Trp | 0.21 | 0.24 | 0.22 | 0.24 | 0.22 |
| Ca | 0.79 | 0.80 | 0.80 | 0.79 | 0.80 |
| TP | 0.66 | 0.69 | 0.69 | 0.70 | 0.72 |

^1^Provided the following per kg of the diet: Cu, 128 mg; Mn, 97.6 mg; Zn, 109 mg; Fe, 197.6 mg; Se, 1 mg; I, 1 mg; Co, 1 mg; VA, 32,500 IU; VD_3_, 10,000 IU; VE, 80 IU; VK_3_, 10 mg; VB_1_, 10 mg/kg; VB_2_, 25 mg; VB_6_, 8 mg; VB_12_, 0.075 mg; biotin, 0.075 mg; folic acid, 5 mg; nicotinamide, 100 mg; pantothenic acid, 50 mg; choline, 1,600 mg; limestone, 0.80%; NaCl, 0.30%; mildewcide, 0.10%; ethoxyquinoline (33%), 0.05%; acidifier, 0.25%.

^2^Calculated according to the nutrient requirements of swine (NRC, 2012); CP, crude protein; ME, metabolic energy; SID, standard ileal digestible; TP, total phosphorus.
