# Supplementary material for: Crude protein content in diets associated with intestinal microbiome and metabolome alteration in Huanjiang mini-pigs during different growth stages
Source: Front Microbiol. 2024 Apr 16;15:1398919. doi: 10.3389/fmicb.2024.1398919 (PMC11058986; doi:10.3389/fmicb.2024.1398919)
Supplement: Supplementary file 4 [file Table_3.DOCX]

**Table S3** Ingredients and composition of the basal diet (air-dried, 20−30 kg growth stage)

| **Items** | **Dietary CP levels (%)** | | | | |
| --- | --- | --- | --- | --- | --- |
|  | **10** | **12** | **14** | **16** | **18** |
| **Ingredients, %** |  |  |  |  |  |
| Corn | 69.65 | 64.25 | 58.65 | 53.35 | 47.60 |
| Soybean meal (43.9%) | 1.00 | 6.90 | 13.20 | 19.10 | 25.50 |
| Soybean meal (fermented) | 2.00 | 2.00 | 2.00 | 2.00 | 2.00 |
| wheat bran | 12.00 | 12.00 | 12.00 | 12.00 | 12.00 |
| Rice hull and bran^1^ | 5.00 | 5.00 | 5.00 | 5.00 | 5.00 |
| Soybean oil | 3.00 | 2.80 | 2.60 | 2.30 | 2.10 |
| L-Lys (98.5%) | 0.95 | 0.80 | 0.60 | 0.45 | 0.25 |
| DL-Met (99%) | 0.15 | 0.15 | 0.10 | 0.10 | 0.05 |
| L-Thr (99%) | 0.35 | 0.30 | 0.20 | 0.15 | 0.05 |
| L-Trp (99%) | 0.10 | 0.10 | 0.05 | 0.05 | - |
| Dicalcium phosphate | 1.10 | 1.00 | 0.90 | 0.80 | 0.75 |
| Premix^2^ | 4.70 | 4.70 | 4.70 | 4.70 | 4.70 |
| Total | 100.00 | 100.00 | 100.00 | 100.00 | 100.00 |
| **Nutrient levels^3^** |  |  |  |  |  |
| ME, MJ/kg | 13.27 | 13.23 | 13.18 | 13.12 | 13.06 |
| CP | 10.12 | 12.11 | 14.10 | 16.10 | 18.13 |
| SID Lys | 1.00 | 1.01 | 1.00 | 1.01 | 1.00 |
| SID Met | 0.29 | 0.31 | 0.29 | 0.31 | 0.29 |
| SID Thr | 0.59 | 0.61 | 0.59 | 0.62 | 0.60 |
| SID Trp | 0.17 | 0.20 | 0.18 | 0.21 | 0.19 |
| Ca | 0.67 | 0.67 | 0.66 | 0.66 | 0.67 |
| TP | 0.54 | 0.54 | 0.55 | 0.56 | 0.57 |

^1^Nutrient levels referred to the China Feed Database Information Network Centre (the 29^th^ edition).

^2^Provided the following per kg of the diet: Cu, 128 mg; Mn, 97.6 mg; Zn, 109 mg; Fe, 197.6 mg; Se, 1 mg; I, 1 mg; Co, 1 mg; VA, 32,500 IU; VD_3_, 10,000 IU; VE, 80 IU; VK_3_, 10 mg; VB_1_, 10 mg/kg; VB_2_, 25 mg; VB_6_, 8 mg; VB_12_, 0.075 mg; biotin, 0.075 mg; folic acid, 5 mg; nicotinamide, 100 mg; pantothenic acid, 50 mg; choline, 1,600 mg; limestone, 1%; sucrose, 2%; NaCl, 0.3%; mildewcide, 0.10%; ethoxyquinoline (33%), 0.05%; acidifier, 0.25%.

^3^Calculated according to the nutrient requirements of swine (NRC, 2012); CP, Crude protein; ME, Metabolic energy; SID, Standard ileal digestible; TP, Total phosphorus.
