# Supplementary material for: Crude protein content in diets associated with intestinal microbiome and metabolome alteration in Huanjiang mini-pigs during different growth stages
Source: Front Microbiol. 2024 Apr 16;15:1398919. doi: 10.3389/fmicb.2024.1398919 (PMC11058986; doi:10.3389/fmicb.2024.1398919)
Supplement: Supplementary file 5 [file Table_4.DOCX]

Table S4−S7 can be found under accession number https://doi.org/10.57760/sciencedb.17573.
